# Editorial: Plant adaptation to climate change using genomic selection and high-throughput technologies

**DOI:** 10.3389/fgene.2024.1471995

**Published:** 2024-08-21

**Authors:** Leonardo Alfredo Ornella, Chiara Broccanello, Monica Balzarini

**Affiliations:** ^1^ CubiqFoods SL, Granollers, Spain; ^2^ Department of Biotechnology, University of Verona (UNIVR), Verona, Italy; ^3^ Consejo Nacional de Investigaciones Científicas y Técnicas, Estadística y Biometría, Facultad de Ciencias Agropecuarias, Universidad Nacional de Córdoba, Córdoba, Argentina

**Keywords:** climate change, genomic selection (GS), GWAS, genome-wide association study, omics, plant breeding

Climate change will negatively impact the yields and nutritional quality of staple (major or orphan) crops. Additionally, the uncertainty of climatic phenomena (frequency, intensity) makes it essential to accelerate the development of varieties adapted to new conditions (Owino et al., 2022).

GWAS (Genome-Wide Association Studies) and GS (Genomic Selection) are powerful approaches to investigate marker-trait associations and reduce breeding time and cost. However, the efficiency of these approaches is influenced by heritability and genetic architecture, and they are not always completely successful. Therefore, new methodologies are needed to complement these approaches and achieve objectives in shorter times.

Rapid advances in high-throughput technologies provide opportunities to develop new alternatives for plant breeding. For instance, there is growing evidence that omics data improve the performance of genomic prediction. Moreover, integrating genome and functional omics data with genetic and phenotypic information can lead to discovering genes and pathways responsible for critical agronomic phenotypes.

The massive amount of data generated by the aforementioned methodologies is linked to phenotypes primarily through machine learning and emerging branches such as deep learning. This discipline can handle the dimensionality and complexity of data, translating biological knowledge and omics data into precision-designed plant breeding (although this task cannot always be solved in real time).

The works presented in this Research Topic cover a wide spectrum of solutions to the challenges generated by climate change, and we believe they will be useful to researchers in the area.

The susceptibility of cultivated potato (*Solanum tuberosum*) to drought poses significant challenges for growers, especially in the context of climate change and the increasing frequency of drought episodes. Fofana et al. evaluated a panel of 384 ethyl methanesulfonate-mutagenized diploid potato clones for drought tolerance and plant maturity under field conditions. The results from the genetic structure analysis of the panel showed five main groups and seven subgroups. Using the Genome Association and Prediction Integrated Tool–mixed linear model GWAS statistical model, 34 and 17 significant quantitative trait nucleotides (QTNs) were found to be associated with maturity and drought traits, respectively. Chromosome five carried most of the QTNs, some of which were also detected using the restricted two-stage multi-locus multi-allele-GWAS haploblock-based model, and two QTNs were found to be pleiotropic for both maturity and drought traits. Using the non-parametric U-test, one and three QTNs, with 5.13%–7.4% phenotypic variations explained, showed favorable allelic effects that increase the maturity and drought trait values. The quantitative trait loci (QTLs)/QTNs associated with maturity and drought traits were found co-located in narrow (0.5–1 kb) genomic regions with 56 candidate genes playing roles in plant development, senescence, and abiotic stress responses. A total of 127 potato clones were found to be late maturing and tolerant to drought, while nine were early to moderate–late maturing and tolerant to drought. Taken together, the data show that the studied germplasm panel and the identified candidate genes are prime genetic resources for breeders and biologists in conventional breeding and targeted gene editing as climate adaptation tools.

Dry beans are a nutrient-dense food targeted in biofortification programs to increase seed iron and zinc levels. The underlying assumption of breeding for higher mineral content is that enhanced iron and zinc levels will deliver health benefits to the consumers of these biofortified foods. Izquierdo et al. characterized a diversity panel of 275 genotypes of *Phaseolus vulgaris* L., comprising the Yellow Bean Research Topic (YBC), for seed Fe and Zn concentration, Fe bioavailability (FeBio), and seed yield across 2 years in two field locations. The genetic architecture of each trait was elucidated via genome-wide association studies (GWAS), and the efficacy of genomic prediction (GP) was assessed. Moreover, 82 yellow breeding lines were evaluated for seed Fe and Zn concentrations as well as seed yield, serving as a prediction set for GP models. Large phenotypic variability was identified in all traits evaluated, with variations of up to 2.8 and 13.7-fold for Fe concentration and FeBio, respectively. Prediction accuracies in the YBC ranged from a low of 0.12 for Fe concentration to a high of 0.72 for FeBio, with an accuracy improvement of 0.03 observed when a QTN identified through GWAS was used as a fixed effect for FeBio. This study provides evidence of the lack of correlation between FeBio estimated *in vitro* and Fe concentration, highlighting the potential of GP in accurately predicting FeBio in yellow beans, offering a cost-effective alternative to traditional assessment methods using Caco2 cell methodologies.

*Eucalyptus dunnii* is one of the most important *Eucalyptus* species for short-fiber pulp production in regions where other species of the genus are affected by poor soil and climatic conditions. In this context, *E. dunnii* holds promise as a resource to address and adapt to the challenges of climate change. Aguirre et al. evaluated the performance of two single nucleotide polymorphism (SNP) genotyping methods for population genetics analysis and Genomic Selection in *E. dunnii*. Double digest restriction-site associated DNA sequencing (ddRADseq) was compared with the EUChip60K array in 308 individuals from a provenance-progeny trial. The compared SNP set included 8,011 and 19,008 informative SNPs distributed along the 11 chromosomes, respectively. Although the two datasets differed in the percentage of missing data, genome coverage, minor allele frequency, and estimated genetic diversity parameters, they revealed a similar genetic structure, showing two subpopulations with little differentiation between them and low linkage disequilibrium. GS analyses were performed for eleven traits using Genomic Best Linear Unbiased Prediction (GBLUP) and a conventional pedigree-based model (ABLUP). Regardless of the SNP dataset, the predictive ability (PA) of GBLUP was better than that of ABLUP for six traits (Cellulose content, Total and Ethanolic extractives, Total and Klason lignin content, and Syringyl and Guaiacyl lignin monomer ratio). When contrasting the SNP datasets used to estimate PAs, the GBLUP-EUChip60K model gave higher and significant PA values for six traits, while the values estimated using ddRADseq gave higher values for three other traits. The PAs correlated positively with narrow sense heritabilities, with the highest correlations shown by the ABLUP and GBLUP-EUChip60K. The two genotyping methods, ddRADseq and EUChip60K, are generally comparable for population genetics and genomic prediction, demonstrating the utility of the former when subjected to rigorous SNP filtering. The results of this study provide a basis for future whole-genome studies using ddRADseq in non-model forest species for which SNP arrays have not yet been developed.

Grass pea (*Lathyrus sativus* L.) is a highly valued legume crop due to its remarkable ability to withstand a variety of environmental conditions, making it a key player in climate-resilient agriculture. Its tolerance to abiotic stresses such as drought, salt, and waterlogging makes it a favored crop in low-input farming systems, especially in South Asia and Sub-Saharan Africa, as well as throughout the Mediterranean Basin. Alsamman et al. performed an identification of bHLH (basic helix-loop-helix) genes in grass pea on a genome-wide scale using genomic and transcriptomic screening. bHLH transcription factors are vital in plant biology, with a significant impact on various aspects of plant growth, cell development, and physiological processes. A total of 122 genes were identified as having conserved bHLH domains and were functionally and fully annotated. The proteins could be classified into 18 subfamilies. There were variations in intron-exon distribution, with some genes lacking introns. The cis-element and gene enrichment analyses showed that the LsbHLHs were involved in various plant functions, including response to phytohormones, flower and fruit development, and anthocyanin synthesis. A total of 28 LsbHLHs were found to have cis-elements associated with light response and endosperm expression biosynthesis. Ten conserved motifs were identified across the LsbHLH proteins. The protein-protein interaction analysis showed that all LsbHLH proteins interacted with each other, and nine of them displayed high levels of interaction. RNA-seq analysis of four Sequence Read Archive (SRA) experiments showed high expression levels of LsbHLHs across a range of environmental conditions. Seven highly expressed genes were selected for qPCR validation, and their expression patterns in response to salt stress showed that LsbHLHD4, LsbHLHD5, LsbHLHR6, LsbHLHD8, LsbHLHR14, LsbHLHR68, and LsbHLHR86 were all expressed in response to salt stress.

Sheath rot disease (SRD) is among the most destructive diseases affecting Manchurian wild rice (MWR) (*Zizania latifolia* Griseb.). In their study, Chen et al. employed a combined transcriptome and metabolome analysis to investigate the responses of Zhejiao No. 7, a cultivar exhibiting some SRD tolerance, to infection. They identified a total of 136 differentially accumulated metabolites (DAMs), with 114 increasing and 22 decreasing in the inoculated plants compared to the control. The up-accumulated metabolites were notably enriched in pathways related to tryptophan metabolism, amino acid biosynthesis, flavonoids, and phytohormone signaling. Transcriptome sequencing revealed 11,280 differentially expressed genes (DEGs), with 5,933 upregulated and 5,347 downregulated in the inoculated plants *versus* the control. The genes involved in tryptophan metabolism, amino acid biosynthesis, phytohormone biosynthesis and signaling, and reactive oxygen species homeostasis supported the metabolite findings. Additionally, genes associated with cell wall structure, carbohydrate metabolism, and plant-pathogen interactions, particularly the hypersensitive response, exhibited altered expression in response to SRD.

In conclusion, by leveraging genome-wide association studies (GWAS) and genomic selection (GS), researchers have been able to accelerate the development of climate-resilient crop varieties, although these methods have limitations that require complementary approaches. Rapid advances in omics technologies and machine learning are critical to translating complex genetic and phenotypic data into actionable insights for precision breeding. This Research Topic spans diverse crops, including potatoes, dry beans, Eucalyptus, grass pea, and Manchurian wild rice, each contributing unique findings to the overarching goal of enhancing climate adaptability. This collective effort underscores the importance of integrating genetic, phenotypic, and environmental data to develop robust, climate-resilient crops, ensuring food security and agricultural sustainability in an unpredictable climate. The insights gained not only advance scientific knowledge but also offer practical solutions for breeders and farmers worldwide.

The integration of diverse methodologies and perspectives not only enriches our understanding but also enhances the applicability of our findings in real-world contexts. As we move forward, it is imperative to continue fostering collaborations across disciplines, prioritizing equitable solutions, and remaining adaptable to emerging trends and technologies. By doing so, we can ensure that our research not only advances academic knowledge but also contributes meaningfully to addressing the pressing Research Topic of our time.
